# Ethnic and sex differences in corneal nerves and corneal epithelial cells in healthy population

**DOI:** 10.3389/fmed.2025.1626501

**Published:** 2025-11-07

**Authors:** Fengyi Liu, Chang Liu, Mingyi Yu, Isabelle Xin Yu Lee, Hassan Mansoor, Ching-Yu Cheng, Wajid Ali Khan, Jodhbir S. Mehta, Yu-Chi Liu

**Affiliations:** 1 School of Clinical Medicine, University of Cambridge, Cambridge, United Kingdom; 2 Regenerative Therapy Group, Singapore Eye Research Institute, Singapore, Singapore; 3 Al-Shifa Trust Eye Hospital, Rawalpindi, Pakistan; 4 Ophthalmology and Visual Sciences Academic Clinical Program, Duke-NUS Medical School, Singapore, Singapore; 5 Epidemiology Group, Singapore Eye Research Institute, Singapore, Singapore; 6 Department of Cornea and External Eye Disease, Singapore National Eye Centre, Singapore, Singapore; 7 Department of Ophthalmology, National Taiwan University Hospital, Taipei, Taiwan

**Keywords:** corneal nerve, corneal epithelial cell, ethnicity, sex, *in-vivo* confocal microscopy

## Abstract

**Purpose:**

We aim to investigate the ethnic and sex differences in corneal nerves and epithelial cell metrics among healthy Chinese and Indian populations.

**Methods:**

This cross-sectional study included 15,850 corneal nerve and 9,510 corneal epithelial cell images from 328 individuals, categorized into Chinese and Indian ethnicities. *In-vivo* confocal microscopy scans were performed to evaluate corneal nerves and epithelium. Quantitative analytic software was used to obtain 10 corneal nerve and epithelial parameters.

**Results:**

There were 208 Chinese participants (101 males, 107 females) in this study with a mean age of 57.0 ± 15.6 years, and 120 Indian participants (58 males, 62 females) with a mean age of 55.8 ± 21.2 years (*p* = 0.36). Compared to Chinese participants, Indian participants exhibited significantly higher values in all nerve parameters, including corneal nerve fiber length, fiber density, branch density, total branch density, fiber area (all *p* < 0.001), fiber width (*p* = 0.041), and fiber fractal dimension (*p* < 0.001). Chinese participants demonstrated significantly larger epithelial size compared to their Indian counterparts (*p* < 0.001). Within the Chinese cohort, females presented with significantly higher corneal nerve fiber length, fiber area, and fractal dimension than males (*p* = 0.034, *p* = 0.022 and *p* = 0.033, respectively). Indian females showed higher epithelial cell circularity compared to Indian males (*p* = 0.026).

**Conclusion:**

Our study identifies significant ethnic and sex disparities in corneal nerves and epithelium. These should be considered when evaluating corneal metrics in Chinese and Indian populations.

## 1 Introduction

The cornea is one of the most highly innervated tissues in the human body, where corneal nerves are necessary for sensations of touch, pain, and temperature, as well as ocular surface maintenance by exerting trophic influences on the corneal epithelium (1). The corneal epithelium, consisting of basal cells, wing cells, and superficial cells connected by desmosome tight junctions (2), serves as the first line of protection against external assaults (3). Corneal nerves and the corneal epithelium mutually support one another to maintain ocular surface homeostasis, with both secreting neurotrophic factors that aid repair and regeneration (4). The corneal epithelium derives innervation from the subbasal corneal nerve plexus which releases epitheliotropic substances, including neurotransmitters and neurotrophic factors (5). Neurotransmitters such as substance P (SP) have been shown to promote epithelial wound healing by stimulating adhesion, migration, and proliferation of epithelial cells (6). Similarly, neurotrophic factors such as nerve growth factor (NGF) support the self-renewal of corneal epithelial progenitor cells and promote epithelial cell survival, proliferation, and migration (7). Reciprocally, some neurotrophic factors derived from corneal epithelium facilitate corneal nerve regeneration once wounded and maintain proper nerve fiber distribution (4).

Some ocular structures have shown ethnicity or sex-related variations in several parameters. For example, South Asian patients with type 2 diabetes were reported to have higher corneal nerve fiber length (CNFL) and corneal nerve branch density (CNBD) compared to European patients, suggesting that South Asians with type 2 diabetes may have a lower risk of developing small fiber neuropathy. Such inter-ethnicity differences in corneal nerve fiber structure may be due to variables like lower levels of smoking and triglycerides, as well as lower BMI in South Asian diabetic patients (8). Moreover, the normative reference values for corneal nerve parameters differ, among published studies that included populations of different ethnic backgrounds (9–11). Besides nerve parameters, it has also been reported that central cornea thickness (CCT) readings are thinner in African Americans compared to Caucasians, while among Chinese, Malays, and Indians, the Chinese have the thickest CCT (12–14). Some suggested explanations for these ethnic variations include genetic predisposition (15, 16). In fact, several CCT-influencing loci were identified in mice, demonstrating a quantitative multigenic pattern of CCT inheritance (17). Ethnic differences in corneal endothelial cell density have also been previously reported, where endothelial cell density is higher in Asians compared to non-Asians and higher in Hispanics compared to non-Hispanics (18, 19).

Sex-related differences have been studied in both murine and human cohorts. Female mice have a faster corneal nerve regeneration rate compared to male mice following corneal injuries. This may be due to an elevated level of brain-derived neurotrophic factor (BDNF) and NGF, in tears secreted in female mice (20). However, conflicting results have been reported in clinical studies where some found higher CNFL, CNBD, and corneal nerve center density (CNFD) in females than in males, while others reported that CNBD and corneal nerve total branch density (CTBD) were higher in males compared to females. Such sex divergence was proposed to be due to hormonal differences or lifestyle differences such as smoking (21, 22).

The potential ethnic-, and sex- divergence in corneal nerve and epithelium parameters may introduce methodological biases if standardized databases developed from one population were applied to a different one. Hence, it is important to develop ethnicity- and sex-adjusted normative databases by examining the normal characteristics as well as the population variations (23). These databases would allow the elimination of ethnic or sex effects from pathological deterioration on various corneal parameters, leading to more accurate detections of subtle, early changes to the corneal nerve and corneal epithelial cell parameters in different populations. Such information would then further facilitate the development of evidence-based medicine for early diagnosis and targeted treatment for different ethnic or sex groups (8).

In this study, we aimed to investigate the ethnic and sex differences in the metrics of corneal nerve and epithelium in Chinese and Indian populations.

## 2 Materials and methods

### 2.1 Study population

This is a cross-sectional, observational study involving 328 healthy individuals and 634 eyes in total. A bi-ethnic cohort consisting of participants of Chinese and Indian ethnicity was recruited from the Singapore Eye Research Institute and Singapore National Eye Center. Sex was recorded as per patients’ medical records, reflecting the information documented at the time of enrollment. The exclusion criteria include conditions that may affect corneal nerve or epithelial status: history of corneal or ocular surface pathology such as keratitis, prior corneal or ocular surgery, glaucoma requiring anti-glaucoma medications, active ocular surface diseases including moderate or severe dry eye disease, current or prior contact lens use >1 year, ongoing intraocular inflammation, diabetes mellitus, and systematic autoimmune diseases (24). Patients were grouped based on their ethnicity and sex. Approval for this study was granted by the Institutional Review Board of SingHealth, Singapore (reference number: 2020/2050), and all procedures strictly adhered to the principles outlined in the Declaration of Helsinki.

### 2.2 *In-vivo* confocal microscopy scans

The examination was carried out using a laser confocal scanning microscope, the Heidelberg HRT3 Rostock Cornea Module (Heidelberg Engineering GmbH, Heidelberg, Germany), with the established protocol (25) by a single, masked and experienced ophthalmologist (CL). The gel-coated objective tip was slowly moved towards the patient to establish contact with the cornea. Participants were instructed to use their contralateral eye to fixate on the blinking light of the microscope, which helps stabilize the view during the scanning process. Both lateral and axial adjustments were made to ensure proper positioning of the objective tip. The cornea was first scanned in its central region, followed by nasal, temporal, superior, and inferior regions, each at a distance approximately 3 mm away from the corneal apex. Every patient had both corneas scanned in this manner. For each subject, IVCM scans were performed to capture the full corneal thickness from epithelium to endothelium across five regions (central, superior, inferior, nasal, and temporal). Sequence scan mode was used, enabling continuous dynamic imaging at a specified depth with data recorded as a video. Each video was automatically converted into individual images (400 × 400 μm, 384 × 384 pixels), with each eye typically yielding 8–10 movies corresponding to 700–900 single images spanning the full corneal thickness.

### 2.3 Image analysis of corneal nerves and epithelium

**Figure 1:**
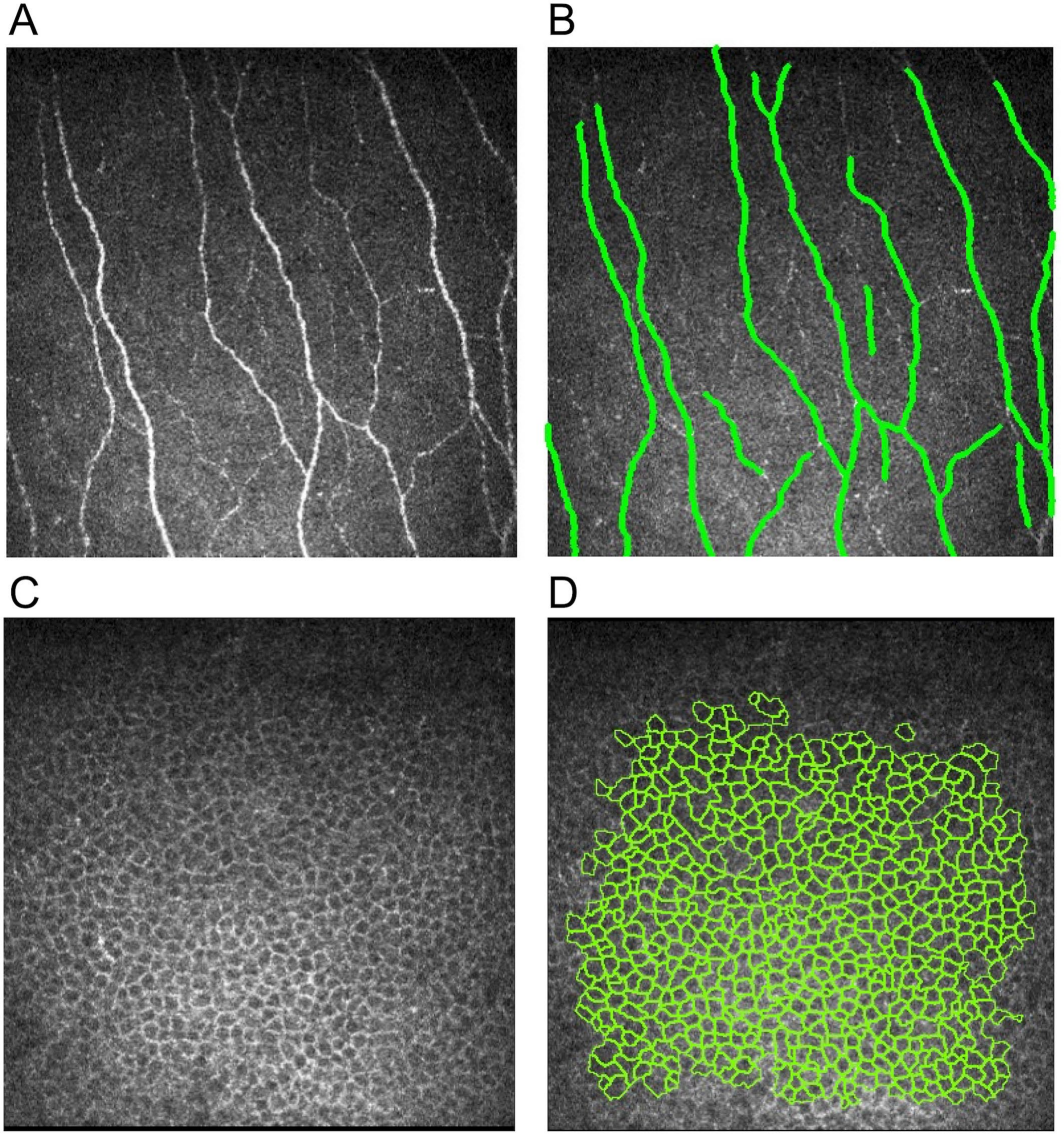
Illustrations of corneal nerve and epithelial cell analysis. **(A)** Raw nerve image, **(B)** nerve image annotated by ACCMetrics software, **(C)** raw corneal epithelial cell image, and **(D)** epithelial cell image with cell annotation by AIConfocal Rapid Image Evaluation System.

The corneal nerve and epithelium images were analyzed with the previously established protocol (26, 27) by an independent, masked and experienced ophthalmologist (MYY). For corneal nerve image analysis, five best-focused and most representative images of subbasal nerves were selected from each of the five areas for both eyes at different depths in a masked and randomized fashion: central, superior, inferior, nasal, and temporal quadrants. A trained and experienced ophthalmologist (CL) used the course of main nerve trunks as landmarks so that each corneal nerve was included only once to avoid overlap in the selected images. In total, 25 images were selected for each eye. These images were analyzed using ACCMetrics software (University of Manchester, Manchester UK) with the following seven nerve metrics being quantified (Figures 1A,B) (28): CNFL (the total length of nerve fibers in mm/mm^2^), CNFD (number of main nerve fibers/mm^2^), CNBD (number of branch points on the main fibers/mm^2^), CTBD (total number of branch points/mm^2^), corneal nerve fiber area (CNFA; total nerve fiber area mm^2^/mm^2^), corneal nerve fiber width (CNFW; average nerve fiber width in mm/mm^2^); and corneal nerve fractal dimension (CNFrD). CNFrD represents the spatial loss of nerves, with a high CNFrD value indicating an evenly distributed complex nerve fiber structure that is more likely to be found in a healthy individual (29) Following quantification, the mean value of the 25 images from each eye was calculated for each of the seven metrics.

For corneal epithelial cell image analysis, the three best images with clear basal cell borders and a large area of coverage were selected from the five areas of the cornea (central, superior, inferior, nasal, and temporal) for each eye. In total, 15 images were selected for each eye. The image selection criteria include great clarity of the epithelial border and large coverage of epithelial cells within the image. Epithelial cell metrics were then quantified by an automated software, AIConfocal Rapid Image Evaluation System (ARIES; ADCIS, France). The following three parameters were obtained: cell density (cells/mm^2^), average size (μm), and circularity (Figures 1C,D). Manual post-processing was performed in ARIES to correct for errors by deselecting non-epithelial cells and outlining any missed epithelial cell borders. These processes were carried out by two independent, trained and experienced ophthalmologists (CL, MYY). The mean value of the 15 images from each eye was calculated for each of the three parameters.

### 2.4 Statistical analysis

The data were expressed as mean ± SD. The normality of the data was assessed using Kolmogorov–Smirnov test. The average of the right and left eye of a patient was used for statistical analysis. A comparison between the two ethnic groups as well as a comparison between the two sexes were carried out using an independent *t-*test. The required sample size was calculated based on the pilot data of 20 patients with the primary outcome, which is CNFD as it has been shown the most reliable among corneal nerve parameters (28) The mean CNFD was 14.5 ± 5.0 and 17.3 ± 5.6 for the Chinese and Indian groups, respectively. Hence, a sample size of 58 patients, with a power of 80% and a significance of 5%, was sufficient to detect the difference between the two ethnic groups. Statistical significance was defined as *p* ≤ 0.05. Statistical analysis was conducted using STATA 17 (STATACorp LP, College Station, TX).

## 3 Results

### 3.1 Patients’ characteristics

A total of 328 participants, comprising 208 Chinese participants (101 males, 107 females) and 120 Indian participants (58 males, 62 females) were included. A total of 15,850 corneal nerve images and 9,510 corneal epithelial images were analyzed. Chinese participants had a mean age of 57.0 ± 15.6 years, while Indian participants had a mean age of 55.8 ± 21.2 years (*p* = 0.36). The mean age of Chinese male and female participants are 57.5 ± 16.0 years and 56.5 ± 15.1 years, respectively, (*p* = 0.43). The mean age of Indian male and female participants is 48.6 ± 21.0 years and 61.8 ± 19.6 years, respectively, (*p* < 0.001). As there was a significant difference in the mean age between Indian males and females, the age-adjusted means obtained from a multivariable regression model were used to compare the nerve and epithelial parameters of these two subgroups.

### 3.2 Comparison of corneal nerve parameters in different ethnic groups

**Figure 2:**
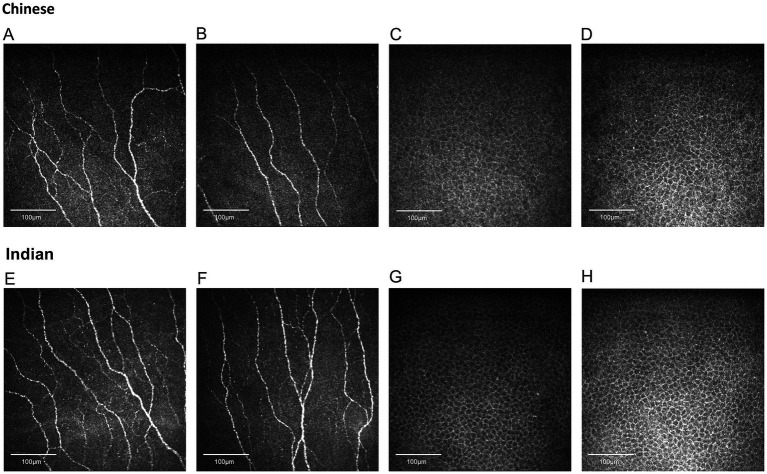
Representative corneal nerve and epithelial cell images for Chinese and Indian participants matched for age and sex. **(A,B)** Nerve images for Chinese, **(C,D)** epithelial cell images for Chinese, **(E,F)** nerve images for Indians, **(G,H)** epithelial cell images for Indians. Indian individuals demonstrate significantly higher values in corneal nerve fiber length, fiber density, branch density, total branch density, fiber area, fiber width, and fiber fractal dimension. Chinese participants have significantly larger epithelial sizes.

**Figure 3:**
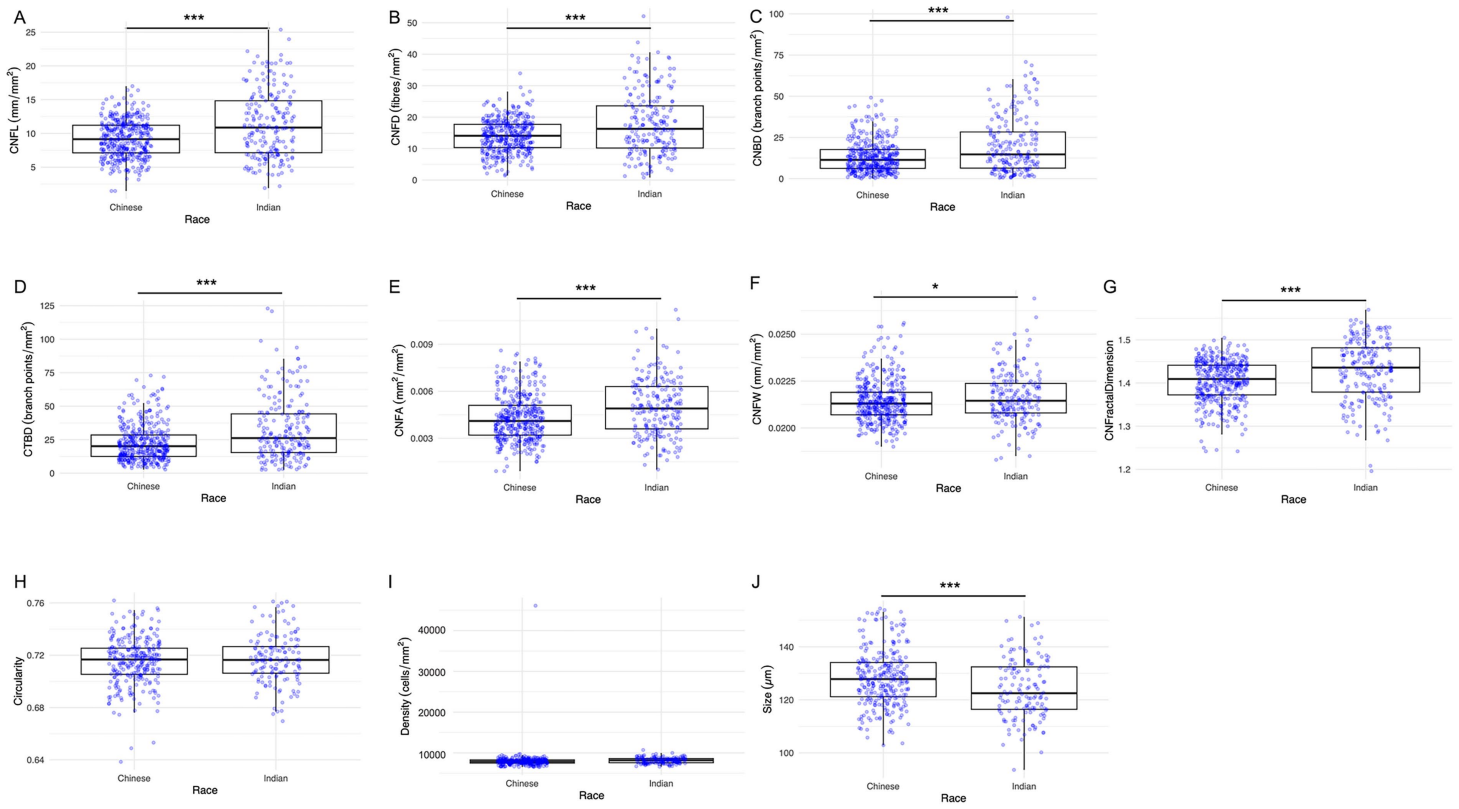
Box and whisker images showing ethnic differences in corneal nerve and epithelial parameters. **(A)** CNFL, **(B)** CNFD, **(C)** CNBD, **(D)** CTBD, **(E)** CNFA, **(F)** CNFW, **(G)** CNFrD, **(H)** Epithelial cell circularity, **(I)** Epithelial cell density, and **(J)** Epithelial cell size. The lower and upper boundaries of the box show the lower quartile (Q1) and the upper quartile (Q3) range of the data, horizontal line within the box indicating the median (Q2). Outliers beyond the range are depicted as dots. Asterisks indicate level of statistical significance: *p < 0.05, **p < 0.01, ***p < 0.001.

The results of the seven nerve parameters assessed for the two ethnic groups are presented in Table 1. Indian participants were found to have denser and longer corneal nerves compared to Chinese participants (Figure 2). Compared to the Chinese, the Indian group exhibited significantly higher values in all nerve parameters: CNFL (*p* < 0.001), CNFD (*p* < 0.001), CNBD (*p* < 0.001), CTBD (*p* < 0.001), CNFA (*p* < 0.001), CNFW (*p* = 0.041), and CNFrD (*p* < 0.001). The corresponding box and whisker plots demonstrating the difference in various nerve parameters between the two ethnic groups are shown in Figure 3.

**Table 1:** Corneal nerve and epithelial parameters in Chinese and Indian groups.

	CNFL (mm/mm^2^)	CNFD (fibers/mm^2^)	CNBD (branch points/mm^2^)	CTBD (branch points/mm^2^)	CNFA (mm^2^/mm^2^)	CNFW (mm/mm^2^)	CNFrD	Circularity	Cell density (cells/mm^2^)	Average size (μm)
Chinese group	9.2 ± 2.7	14.1 ± 5.2	13.1 ± 9.3	22.5 ± 13.4	0.00428 ± 0.0014	0.0214 ± 0.0011	1.40 ± 0.05	0.715 ± 0.018	8,007 ± 2,367	128.2 ± 10.5
Indian group	11.4 ± 4.9	17.8 ± 9.8	20.6 ± 17.5	32.7 ± 23.2	0.00499 ± 0.00186	0.0216 ± 0.0013	1.43 ± 0.07	0.718 ± 0.019	8,146 ± 731	124.0 ± 11.1
*p* value	**<0.001**	**<0.001**	**<0.001**	**<0.001**	**<0.001**	**0.041**	**<0.001**	0.289	0.483	**<0.001**

Bold values indicate statistical significance (*p* < 0.05).

### 3.3 Comparison of corneal epithelial parameters in different ethnic groups

The epithelial cells are homogenous, regular, and densely packed with well-defined cell boundaries and small cell bodies, showing no morphological abnormalities (Figure 2). Indian participants had significantly lower epithelial size compared to Chinese participants (Figure 3) (*p* < 0.001). On the other hand, the other two parameters, circularity and cell density, did not show significant differences between the two ethnic groups. Table 1 and Figure 3 present the results of the three epithelial parameters assessed for the two ethnic groups.

### 3.4 Comparison of corneal nerve and epithelial parameters in different sex groups

**Figure 4:**
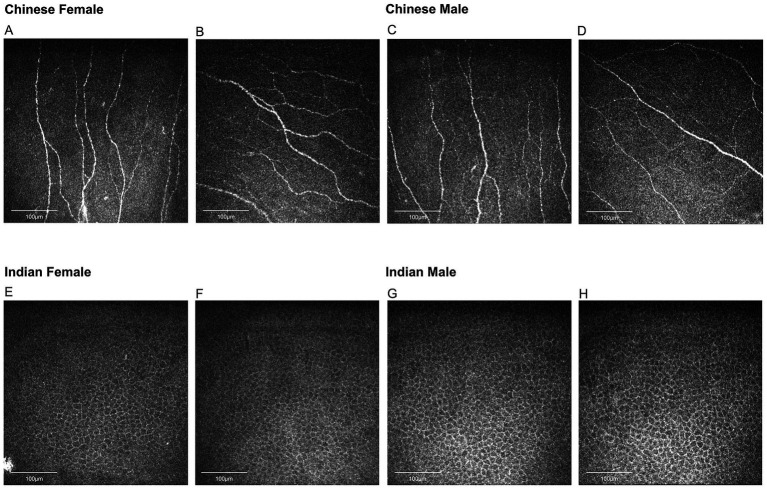
Representative corneal nerve images for Chinese participants and epithelial cell images for Indian participants. **(A,B)** Nerve images for Chinese females, **(C,D)** nerve images for Chinese males, **(E,F)** epithelial cell images for Indian females, **(G,H)** epithelial cell images for Indian males. Chinese females have significantly higher corneal nerve fiber length, fiber area, and fractal dimension than Chinese males. Indian females have higher epithelial cell circularity compared to Indian males.

Table 2 represents the detailed results of nerve and epithelial parameters assessed for the two sex groups within each ethnic group. Significant sex-dependent differences in corneal nerve parameters were observed among Chinese participants. Specifically, three corneal parameters were significantly higher in female Chinese participants compared to their male counterparts: CNFL (*p* = 0.034), CNFA (*p* = 0.022), and CNFrD (*p* = 0.033) (Figure 4). For the Indian group, Indian female has higher age-adjusted means for epithelial cell circularity than Indian male participants (*p* = 0.026) (Figure 4).

**Table 2:** Corneal nerve and epithelial parameters sorted by ethnicity and sex.

Chinese ethnic group										
	CNFL (mm/mm^2^)	CNFD (fibers/mm^2^)	CNBD (branch points/mm^2^)	CTBD (branch points/mm^2^)	CNFA (mm^2^/mm^2^)	CNFW (mm/mm^2^)	CNFrD	Circularity	Cell density (cells /mm^2^)	Average size (μm)
Male	8.9 ± 2.8	13.6 ± 5.4	12.5 ± 9.1	21.2 ± 12.9	0.00411 ± 0.00141	0.0214 ± 0.0011	1.40 ± 0.05	0.715 ± 0.019	8,133 ± 3,333	128.5 ± 10.9
Female	9.5 ± 2.6	14.5 ± 4.9	13.8 ± 9.5	23.8 ± 13.8	0.00444 ± 0.00146	0.0215 ± 0.0011	1.41 ± 0.05	0.715 ± 0.016	7,887 ± 616	128.0 ± 10.2
*p* value	**0.034**	0.096	0.164	0.053	**0.022**	0.468	**0.033**	0.947	0.385	0.679

Indian ethnic group age-adjusted means										
	CNFL (mm/mm^2^)	CNFD (fibers/mm^2^)	CNBD (branch points/mm^2^)	CTBD (branch points/mm^2^)	CNFA (mm^2^/mm^2^)	CNFW (mm/mm^2^)	CNFrD	Circularity	Cell density (cells/mm^2^)	Average size (μm)
Male	11.4	18.0	20.2	32.6	0.00488	0.0215	1.43	0.714	8,086	123.0
Female	11.0	16.7	19.0	30.0	0.00489	0.0218	1.43	0.721	8,077	125.1
*p* value	0.409	0.250	0.571	0.320	0.935	0.201	0.874	**0.026**	0.952	0.283

Bold values indicate statistical significance (*p* < 0.05).

## 4 Discussion

In this study, we demonstrated ethnic variation in corneal nerve and epithelial parameters, revealing significantly higher CNFL, CNFD, CNBD, CTBD, CNFA, CNFW, CNFrD, and significantly smaller cell size of corneal epithelial cells in Indian participants compared to their Chinese counterparts. We also showed sex-based differences in corneal innervation and epithelial cells among Chinese and Indian participants. Chinese females demonstrated significantly higher CNFL, CNFA, and CNFrD than Chinese males, while Indian females showed significantly higher cell circularity compared to Indian males. This study highlights the importance of investigating ethnic and sex differences in physiological and pathological features amidst global healthcare diversification and increasing social-ethnic diversity. Our findings would help establish an ethnicity- and sex-adjusted normative database.

IVCM has been used extensively and reliably to image corneal nerve and corneal epithelial cells *in vivo*, assessing both their physiological morphology and pathological alterations (26, 28, 30, 31). For instance, the alterations in corneal nerve metrics have been shown in dry eye disease, diabetic corneal neuropathy, neurotrophic keratopathy, and Sjögren’s syndrome (32, 33). Similarly, morphological changes or cell loss in the corneal epithelium were also observed in a range of corneal or systemic diseases, such as diabetes, recurrent corneal erosions, or lattice corneal-dystrophy (34, 35). Hence, an accurate database is essential for better guiding the diagnosis and monitoring the disease progression. However, ethnicity and sex present a challenge in this area. Notably, though Chinese and Indian populations are predominant in Singapore, there is a lack of comprehensive comparison between these two populations in terms of corneal nerve and epithelial status, leaving the inter-ethnic disparity unaccounted for.

To our knowledge, this study is the first to demonstrate the structural difference of corneal nerves between Chinese and Indian populations in healthy subjects. Fadavi et al. (8) found that CNFL and CNBD are significantly higher in South Asian diabetic patients compared to European diabetic patients. Zhang et al. (36) reported that Mongolian diabetic patients have higher CNFD, CNFL, and CNBD compared to Han diabetic patients. Advancing age has been reported to affect both corneal nerve and epithelial parameters (26) However, there is no significant difference between the mean age of the two ethnic groups in the present study. We found that Indian ethnic groups in this study exhibited significantly higher morphological corneal nerve metrics compared to the Chinese ethnic group. Corneal nerve morphology and functions are mediated by various neuromediators, cytokines/chemokines, and hormonal regulators (37) Inter-ethnic genetic variation affecting the expression of these modulators may primarily contribute to the observed divergence of corneal nerve parameters between the two ethnic groups. Differing polymorphisms of some neuromodulator genes between Chinese and Indians might account for the varying levels of nerve status. Interleukin-6 (IL-6) is a neuropoietic cytokine responsible for immunomodulation, neuronal survival and function. Gan et al. demonstrated that the frequency of IL-6174 (G/C) polymorphism was 19.0% in the Indian population while it was not detected in the Chinese population (38) In addition to IL-6, vascular endothelial growth factor (VEGF) has been shown to contribute to neuron growth and neuroprotection. A murine study demonstrated that VEGF accelerated corneal nerve growth, resulting in not only a rapid return of corneal sensation but also neurotrophic effects on the corneal nerves (39) VEGF +405GG genotype was suggested to have a protective role for peripheral nerves (40) Though no direct comparison of VEGF genotype frequency has been made between Chinese and Indian populations, studies conducted in India and China have separately shown that 77.4% of healthy individuals of the South Indian ethnicity have the VEGF +405GG genotype, while only 35.2% of healthy individuals of Chinese Han origin have the VEGF +405GG genotype (41, 42) Hence, higher levels of IL-6 and VEGF in the Indian population may explain their greater nerve fiber density, length, area, width, branch density, and the more uniform distribution of their corneal nerves.

Dietary and lifestyle choices may also explain the observed inter-ethnic difference in corneal nerves. Neurotrophins from phytochemicals in Indian food preparations may contribute to Indians’ higher corneal nerve parameters. Curcumin, which is commonly used for Indian food flavoring, was shown to be able to induce brain-derived neurotrophic factor (BDNF) and exert anti-neuroinflammatory and neurotogenesis-inducing effects, stimulating nerve growth (43). Besides, a previous study has shown that classical risk factors for coronary heart disease, such as smoking, may account for structural differences in small nerve fibers between South Asians and Europeans (8). Chronic smoking was found to be negatively correlated with total subbasal nerve numbers. The negative impact of smoking on the ocular surface may be directly from the toxic material in cigarette smoke, such as free radicals and highly reactive carbonyls (44). According to the Singapore National Health Surveillance Survey, adult Chinese had a higher daily smoking prevalence (7.9%) compared to Indians (5.9%) (45).

Additionally, we found that Indians have significantly smaller corneal epithelial cell sizes compared to the Chinese. It was suggested that a smaller epithelial cell size may be indicative of higher proliferative activity (46). The basal epithelial cells in the limbus were notably smaller compared to those in the central cornea, which corresponded with the higher proliferative capacity observed in the limbal epithelial cells (47, 48). This is consistent with our data that Indians showed numerically higher corneal epithelial cell density compared to Chinese (8,146 ± 731 cells/mm^2^ versus 8,007 ± 2,367 cells/mm^2^), although this difference was not statistically significant (*p* = 0.483). The corneal nerve plexus contributes to epithelial proliferation by releasing multiple neuromodulators such as epithelial growth factor and ciliary neurotrophic factor (34). Hence, the higher proliferative capacity of epithelial cells in the Indian ethnic group might be due to the better state of the corneal nerve with more neurotrophins secreted, as indicated by the higher nerve parameters in Indians. Ishibashi et al. (49) further reported that corneal epithelial basal cell density is positively and significantly correlated with CNFD and CNBD.

With regard to sex variation of nerve parameters, we found that Chinese females presented with significantly higher CNFL, CNFA, and CNFrD compared to Chinese males. We postulate this is due to hormonal and lifestyle differences. Progesterone has been shown to stimulate neurite outgrowth, accelerate the maturation of the regenerating axons, and promote myelin formation in peripheral nerves (50). This is supported by a murine study where nerves in female rats were found to sprout and regenerate more quickly following injury compared to their male counterpart. This might be due to the effect of estrogen and/or progesterone on proliferating Schwann cells as well as nerve cell bodies (51). Another animal study found that topical application of *β*-estradiol promoted corneal nerve regeneration and increased subbasal nerve density, through enhancing the secretion of BDNF and NGF (20). In addition to hormones, lifestyle choices like smoking may also affect corneal nerve status (44). In a population-based study examining Chinese residing in Singapore, females were found to smoke less than males (52). Similarly, Cao et al. (21) presented that Chinese females have significantly higher CNFD, CNBD, and CNFL compared to their male counterparts. Apart from corneal nerves, sex differences were also observed in intraepidermal fiber density (IENFD) and peripheral nerve size, with studies indicating that males generally have less dense IENFD and larger nerve cross-sectional area in nerves like median, ulnar, and tibial nerves (23, 53, 54). On the other hand, Markoulli et al. (22) reported that males have significantly higher CNBD and CTBD compared to females, which contrasts with our results. This discrepancy may be attributed to the fact that Markoulli et al. (22) only scanned the central cornea of the right eye, whereas we scanned the central and four peripheral quadrants of the corneas of both eyes, providing a more accurate and representative evaluation. Another possible explanation is the different demographics of participants where Markoulli’s study was conducted in Australia while ours was in Singapore (55, 56). Earlier studies reported no significant sex differences in corneal nerve metrics including CNFL, CNBD, CNFD, and corneal nerve fiber tortuosity (CNFT) (57–59). Such disparity may arise because the earlier studies only included three to eight nerve images of the central corneal region per eye, while our study included 25 images per eye, representing more comprehensive nerve status over the whole cornea.

Besides nerve parameters, sex variation was also observed in corneal epithelial cell parameters. We found that Indian females have significantly higher cell circularity compared to Indian males. Throughout the corneal epithelial regeneration, the newly regenerated basal epithelial cells are ovoid in shape. As these cells mature, they gradually become more cuboidal and more columnar (26). A higher cell circularity may therefore indicate a higher regeneration capacity in Indian females as they have a higher percentage of newly regenerated ovoid-shaped epithelial cells. Such observation may be explained by higher expression of genes that regulate cell proliferation in female corneal epithelial cells, for example, guanine nucleotide binding protein-like 3 (nucleolar) (60). Similar findings have also been reported in in-vitro intestinal epithelial stem cells (IESCs) where the growth of IESCs in females was enhanced compared with males (61).

Our study has several limitations. Firstly, we only included Chinese and Indian populations. In future studies, the inclusion of other ethnic groups would provide additional data across diverse ethnic demographics. Secondly, we did not investigate the underlying molecular basis accounting for these observations. However, the main aim of this study was to understand if ethnic differences exist in corneal nerve and epithelium metrics. Subsequent studies shall aim to elucidate the mechanism causing the inter-ethnic difference in corneal nerve and epithelium status. Thirdly, this study is based on self-reported ethnicity. Future research could benefit from incorporating genetic measures of ancestry to determine the ethnic profiles of the participants more accurately. Fourthly, nerve metrics assessment was performed using ACCMetrics across both central and peripheral corneal images. Given that the tool is validated primarily for central images, its performance in peripheral areas – where nerve architecture is more complex – may be less reliable. Nevertheless, it remains the most widely used and accepted tool for automated corneal nerve analysis. Future studies would benefit from applying methods validated specifically for the peripheral cornea and reporting central and peripheral data separately. Lastly, smoking status was not systematically collected, hence confounding by tobacco exposure cannot be excluded (62). Future studies should obtain standardised smoking histories and incorporate these variables into adjusted analyses.

In conclusion, our findings indicate that Indian participants have corneal nerve fibers that are denser, wider, longer, more uniformly distributed, and healthier than those of Chinese participants. Additionally, the corneal epithelial cells in Indians are smaller compared to those in Chinese. We also showed that Chinese females have higher CNFL, CNFA, and CNFrD compared to their male counterparts while Indian females have higher epithelial cell circularity compared to Indian males. The identification of ethnic- and sex-divergence in corneal nerve and epithelium would help improve the diagnostic accuracy of corneal and associated diseases, providing valuable reference levels across ethnicities and sexes. Our study was designed to define such ethnic- and sex-specific normative variation in healthy participants. The observed variation may bias disease studies if unaccounted for, especially when using threshold-based cut-offs. Adjusting analyses for ethnicity and sex or using ethnicity−/sex-adjusted reference ranges, may therefore help prevent spurious diagnostic or treatment-response findings driven by demographic factors rather than true biological effects.

## Data Availability

The raw data supporting the conclusions of this article will be made available by the authors, without undue reservation.
